# Stability Assessment of Candidate Reference Genes in Urine Sediment of Prostate Cancer Patients for miRNA Applications

**DOI:** 10.1155/2015/973597

**Published:** 2015-05-20

**Authors:** Maria Giulia Egidi, Giovanni Cochetti, Gabriella Guelfi, Danilo Zampini, Silvana Diverio, Giulia Poli, Ettore Mearini

**Affiliations:** ^1^Department of Surgical and Biomedical Sciences, Institution of Urological, Andrological Surgery and Minimally Invasive Techniques, University of Perugia, Loc. S. Andrea delle Fratte, 06156 Perugia, Italy; ^2^Department of Veterinary Medicine, University of Perugia, Via San Costanzo 4, 06126 Perugia, Italy

## Abstract

We aimed at assessing the stability of candidate reference genes in urine sediments of men subjected to digital rectal examination for suspected prostate cancer (PCa). Two microRNAs (miR-191 and miR-25) and 1 small nucleolar RNA (SNORD48) were assayed in 35 post-DRE urine sediments of men with PCa and in 26 subjects with histologically confirmed benign prostatic hyperplasia (BPH). The stability of candidate reference genes was assessed through BestKeeper algorithm and equivalence test. miR-200b and miR-452 were used to test for the effect of normalization on target genes. Our results proved miR-191 to be the most stable gene, showing the lowest degree of variation and the highest stability value. miR-25 and SNORD48 values fell beyond the cutoff of acceptability. In conclusion, we recommend the use of miR-191 for normalization purposes in post-DRE urine sediments.

## 1. Introduction

Prostate cancer (PCa) represents the second most common male cancer [1]. In Western countries, the main diagnostics tools for PCa diagnosis are PSA monitoring together with digital rectal examination (DRE). Although the introduction of PSA testing in clinical practice has dramatically improved the early diagnosis and consequently the treatment of localized disease, the real benefits of an extensive use of PSA remain controversial [2]. Since PSA is a prostate-specific and not a cancer-specific marker, there is an urgent need for new biomarkers able to diagnose real disease and not benign conditions which also cause an increase in serum PSA.

MicroRNAs (miRNAs) represent a class of small, noncoding RNAs which targets complementary sites of messenger RNA (mRNA) and negatively regulates their expression [3]. In the last years, microRNAs have attracted scientific interest upon demonstration of their alteration in response to various diseases: they have been proved to affect the crucial steps of carcinogenesis acting as oncogenes or oncosuppressors [4]. Real-time PCR is an extremely versatile tool to analyse gene expression: the selection of stably expressed reference genes is a crucial step which inevitably affects data reliability. Often, contrasting results are attributable to improper normalization approaches which impair the objectivity of data. For large RNAs, the stability of several reference genes has been widely reviewed [5–8] and even called into question [9–11]. For microRNAs (miRNAs), the stability of putative reference genes has not yet been completely confirmed and waits for adequate assessments in various biological matrices, though several attempts have been made so far [12–17] also for prostate cancer [18–20]. Small nucleolar RNAs (snoRNAs) [21] are quite often used to normalize tissue miRNA expression; one such example is represented by the SNORD family, which proved to be stable in several biological matrices [22–24]. In particular, SNORD48 has been often used as reference gene in miRNAs expression studies on prostate cancer tissues [25–27]. Starting from these considerations, the present study was aimed at assessing the stability of miR-25, miR-191, and SNORD48 in post-DRE urine sediments of 35 urine sediments of men undergoing radical prostatectomy for prostate cancer. Voided urine obtained immediately after DRE is greatly enriched in prostatic cells (about 80%) and thus represents a challenging starting material for biomarker discovery in oncological research. 26 healthy subjects with benign prostatic hyperplasia (BPH) subjected to the same clinical maneuver were recruited as controls. To date, there are no reports regarding the stability assessment of these genes in post-DRE urine sediments. The two miRNAs were selected as candidate reference genes on the basis of literature data regarding their stability in several biological matrices [18], whereas SNORD48 was commercially available as a reference control. miR-200b and miR-452 were used to test for the effect of normalization on target genes.

## 2. Materials and Methods

### 2.1. Patients

61 subjects subscribing an informed consent were included in the study. Of these, 35 underwent radical prostatectomy for localized prostate cancer and 26 underwent prostate biopsy for suspected PSA levels. Histological examination of tissue biopsy confirmed the absence of cancer in these subjects. Clinical parameters of subjects enrolled in the study are provided in Table 1.

### 2.2. Post-DRE Urine

First catch voided urine (30 mL) was collected after attentive digital rectal examination and prior to prostate biopsy or surgical intervention. Urine samples were immediately centrifuged (2000 ×g, 10 min, 4°C) and cell pellets were washed twice with phosphate buffered saline. 300 *μ*L lysis and stabilization buffer were added to the pellets and lysates were stored at −80°C until use.

### 2.3. RNA Isolation

Total RNA Extraction Kit (Norgen Biotek Corp., Ontario, Canada) was used to isolate RNA. After extraction, RNA has been subjected to qualitative assessment through Bioanalyzer and NanoDrop. Total RNA was quantified by Qubit RNA Assay (Life Technologies) and stored at −80°C until use.

### 2.4. Reverse Transcription and Real-Time PCR

7.5 ng of total RNA was reverse-transcribed with miRCURY LNA Universal RT microRNA PCR, polyadenylation, and cDNA synthesis kit (Exiqon). 0.5 *μ*L of UniSp6 spike-in control was added to the retrotranscription mix (total volume 10 *μ*L) before incubation as a positive cDNA synthesis control. For real-time PCR amplification, miRCURY LNA specific PCR primer set (Exiqon system) and Exiqon miRCURY LNA Universal RT microRNA PCR SYBR Green master mix were used. Primers were designed based on the mature sequences of genes. Stable Ct values obtained from the amplification of spike-in RNA with LNA control primer set (Exiqon) were used as quality control. Primer sequences, gene symbols, and accession numbers are listed in Additional File 1 available online at http://dx.doi.org/10.1155/2015/973597. Standard curves were generated for each primer using serial dilutions of known quantities of cDNA in triplicate (curves are provided in Additional File 2, together with DNA concentrations used and corresponding efficiences). PCR reactions were performed on a BioRad iCycler (BioRad, Hercules, CA). 5 *μ*L of SYBR Green master mix was mixed with 1 *μ*L of primer mix and 4 *μ*L of diluted cDNA (1 : 10) (total reaction volume: 10 *μ*L). Thermal cycling conditions included 10 min at 95°C for enzyme activation and 45 cycles of amplification (15 sec 95°C for denaturing double stranded DNA and 1 min at 60°C for annealing/extension steps). Melting curve analysis was performed to assess amplification specificity. Each sample was run in triplicate and the results were averaged; no-template controls were included in the analysis. For each sample, LNA control primer set was used to amplify UniSp6 spike-in positive control. All real-time PCR assays meet requirements of the Minimum Information for Publication of Quantitative Real-Time PCR Experiments (MIQE) guidelines [28]. The MIQE checklist is provided in Additional File 3. The 2^−ΔCt^ method was employed to normalize raw Ct data. Mean Cts of miR-191, miR-24, and SNORD48 from BPH group were used as the calibrator sample (e.g., ΔCt miR-25 = Ct miR-25_PCa_ − Ct miR-25_avg,BPH_).

### 2.5. Statistical Analysis

The BestKeeper [29] algorithm was used to assess the stability of candidate reference genes. Mean, standard deviation (SD) and coefficient of variation (CV) of quantification cycles (Cq) were calculated. GraphPad Prism 6.0 (GraphPad Software, Inc., San Diego, CA) was used for descriptive statistics of raw Ct data and to calculate variance between PCa and BPH groups. Equivalence test [30] was performed with XLSTAT (Addinsoft).

## 3. Results

RNA concentrations ranged from 0.5 to 14 ng/*μ*L. No statistically significant difference in RNA yields was observed between PCa and BPH groups (*p* > 0.05). Mean RNA integrity number (RIN) was 8.7 (range 8.3–9.0). 260/280 ratio was 1.84 (range 1.84–2.03).

### 3.1. Expression Levels of Putative Reference Genes

**Figure 1:**
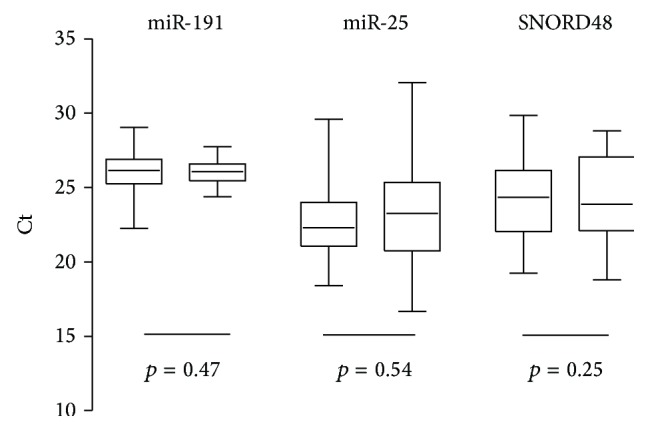
Expression levels (as raw Cts) of miR-191, miR-25, and SNORD48 in post-DRE urine sediments from PCa (left) and BPH (right) subjects. Boxes represent lower and upper quartiles; whiskers represent the minimum and maximum value.

Ct values of miR-25, miR-191, and SNORD48 ranged between 23 and 26 (Figure 1). Mann-Whitney *U* test was performed to verify if candidate genes were differentially expressed in the two groups. No statistically significant differences were observed for all genes under analysis (miR-191, *p* value 0.47; miR-25, *p* value 0.54; SNORD48, *p* value 0.25) (Figure 1).

### 3.2. Relative Expression of Candidate Reference Genes

**Figure 2:**
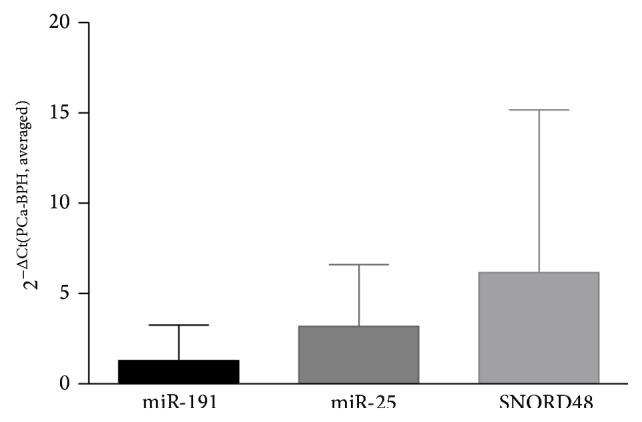
Normalized Ct values of miR-191, miR-25, and SNORD48 in post-DRE urine sediments from PCa patients. Mean Ct values from BPH group served as calibrator sample.

Ct values of miR-191, miR-25, and SNORD48 from PCa patients were normalized using BPH group as calibrator sample. Relative expression was calculated as ΔCt (e.g., ΔCt miR-25 = Ct miR-25_PCa_ − Ct miR-25_avg,BPH_) (Figure 2).

### 3.3. Stability of Putative Reference Genes

**Figure 3:**
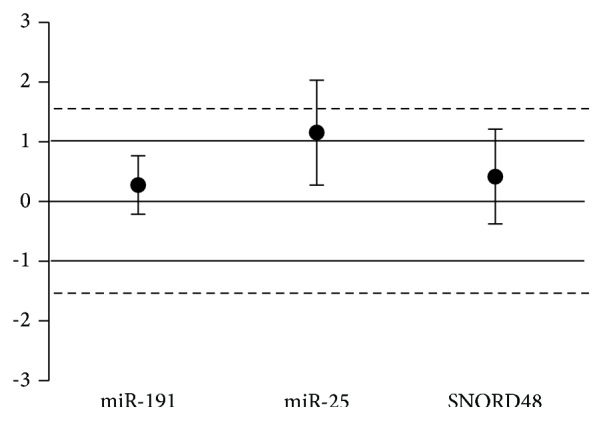
Equivalence test for miR-191, miR-25, and SNORD48 in PCa and BPH groups. Solid circles represent the differences of means and bars indicate symmetrical confidence intervals. Logarithmic expression values were used to calculate the relative expression of each gene in PCa compared to BPH group. Fold changes in expression levels between prostate cancer (PCa) and benign prostatic hyperplasia (BPH) groups are plotted on *y*-axis. Deviation area between solid lines ranging from −1 to 1 corresponds to fold changes ≤ 2, whereas dotted lines indicate a deviation area corresponding to a fold change ≤ 3. Equivalence was satisfied only by miR-191 (difference between means 0.277; CI [−0.07, 0.624]), whereas miR-25 (difference between means 1.155; CI [0.275, 2.035]) and SNORD48 (difference between means 0.419; CI [−0.375, 1.212]) fell out of the cutoff limits.

Stability of selected genes was assayed by means of BestKeeper algorithm. Values are reported in Table 2. Statistical analysis found mir-191 as the most stable gene (0.842), whereas values for miR-25 and SNORD48 exceeded the cutoff of acceptability (2.212 and 5.698, resp.). Equivalence was satisfied only by miR-191 (difference between means 0.277; CI [−0.07, 0.624]), whereas miR-25 (difference between means 1.155; CI [0.275, 2.035]) and SNORD48 (difference between means 0.419; CI [−0.375, 1.212]) fell out of the cutoff limits (Figure 3).

### 3.4. Effect of Normalization on Target Genes

**Figure 4:**
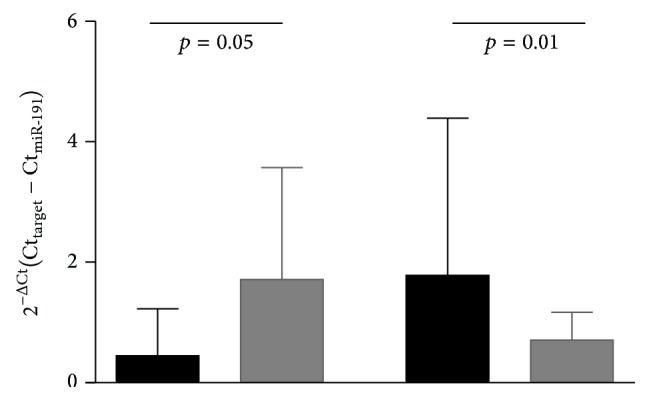
Normalized Ct values of miR-200b and miR-452 using miR-191 as reference gene in PCa (black columns) and BPH (grey columns) subjects. Values are reported as mean ± SEM.

miR-200b and miR-452 were used as target genes to verify the effect of normalization approach on their expression. Results are reported in Figure 4.

## 4. Discussion

Biomarker discovery in oncological research holds promise to improve the early diagnosis of cancer; in this regard, miRNAs have greatly attracted scientific interest because of their established alteration in response to carcinogenesis [4]. Real-time PCR represents the major technique to explore variations in gene expression and the most used strategy is the relative quantification approach [5–8]. Here, the gene of interest is normalized against an endogenous control whose expression remains unaltered in the samples under analysis. The proper choice of an endogenous control is a crucial step which inevitably affects the reliability of scientific results [9–11]. Many scientific works have assessed the stability of several miRNAs in diverse biological matrices for several diseases and thus represent a starting point for those dealing with similar experimental conditions [12–19]. One of the most powerful works evaluating the stability of miRNAs across various tissues was made in 2008 by Peltier and Latham [20]. They analysed the stability of several miRNAs in several normal solid tissues, including miR-191 and miR-25. In this first phase of the study, miR-191 proved to be most consistently expressed gene among almost all healthy tissues. In a second phase of the same study, the authors analysed 5 sets of tumor and normal adjacent tissue pairs to verify the stability of the selected controls upon carcinogenesis. Again, the combined analysis of tumor tissue and normal counterpart showed miR-191 to be the most stable miRNA. Another normalization strategy is the use of exogenous spike-ins: the addition of these miRNAs (primarily cel-miR-39 from* C. elegans*) to the lysis buffer during RNA isolation step would guarantee a proper normalization of PCR data. This approach has been widely used to normalize PCR data of extracellular miRNAs [31–33], although the classical strategy employing reference genes after testing their stability in the sample under analysis is quite often recommended [34] and used [35–37]. While many studies dealt with circulating miRNA profiling as diagnostic tool for urologic diseases, few reports to date delved into their characterization in urine sediment [38–40]. In the present study, we aimed to assess the stability of two microRNAs (miR-191, miR-25) and a small nucleolar RNA (SNORD48) in urine sediment obtained after attentive digital rectal examination. Urine post-DRE is achievable for all men suspected for prostate cancer; thus it represents a minimally invasive and challenging starting material for biomarker discovery. Furthermore, up to 80% of cells of post-DRE urine sediment are of prostatic origin; thus this sample contains enough prostatic cells to make real-time PCR assays feasible. The two miRNAs were chosen on the basis of literature data demonstrating their stability in various tumor tissues compared to healthy counterparts [12, 18, 25]. Our results confirmed the stability of miR-191 in urine sediments, whilst miR-25 and SNORD48 fell beyond the cutoff of acceptability. SNORD48 has been proved to be stably expressed in neoplastic tissues compared to healthy surrounding counterparts [25–27]. Although often stable, a recent study demonstrated the alteration of several snoRNAs, including SNORD48, in many diseases, raising doubt about their reliability as reference genes [41]. Moreover, these products are longer than miRNAs and thus could differ in terms of extraction yield. We choose as target gene miR-200b because it belongs to a family of tumor-suppressive miRNAs and it has been demonstrated to be downregulated in prostate cancer [42–44]. In contrast, miR-452 was proved to be massively upregulated in 6 stem/progenitor cell populations in prostate cancer [45].

## 5. Conclusions

Normalization of miRNAs is a yet complex and fragmented picture, and although many attempts have been made in this direction, a set of housekeeping genes to be used for PCR normalization has not yet been characterized. This is undoubtedly due to the fact that the study of miRNAs is a relatively young field of research; thus reliable data about invariant products to be used for normalization purposes simply need to grow up. Our work demonstrated the stability of miR-191 in post-DRE urine sediments.

## Supplementary Material

Supplementary Material includes a list of primer sequences, together with gene symbols and accession numbers. This information is shown in Additional File 1. Standard curves generated for each primer are shown in Additional File 2; DNA concentrations and efficiencies are reported. All assays meet requirements of the Minimum Information for Publication of Quantitative Real-time PCR Experiments (MIQE) Guidelines; MIQE checklist is provided in Additional File 3.

## Figures and Tables

**Table 1:** Clinicopathological data of subjects involved in the present study.

Clinical parameters	PCa	BPH
Mean age (range)	72 (51–87 ys)	66.5 (48–77 ys)
Mean PSA (range)	6.09 ng/mL (3.16–28.6)	7.44 (1.38–21.2 ng/mL)
Mean prostate volume (range)	53.8 cc (37.5–75.2)	55.6 cc (36.5–80.8)
Clinical stage		—
T1c	15	
T2a	10	
T2b	8	
T2c	2	
Pathological Gleason score		
6 (3 + 3)	18	
7 (3 + 4)	12	
7 (4 + 3)	5	
Pathological stage		
T2a	11	
T2b	15	
T2c	9	

**Table 2:** Analysis of gene stability made through BestKeeper algorithm; Pearson correlation coefficients (left panel) and stability values (right panel).

Pearson correlation coefficient (*r*)				Stability value			
	miR-191	miR-25	SNORD48		miR-191	miR-25	SNORD48
miR-25	0.375 (*p* = 0.003)	—	—	Gene stability	0.842	2.212	5.698
SNORD48	0.157 (*p* = 0.226)	0.207 (*p* = 0.109)	—	BestKeeper versus *r* (*p* = 0.01)	0.616	0.824	0.770
