# Supplementary material for: Stability Assessment of Candidate Reference Genes in Urine Sediment of Prostate Cancer Patients for miRNA Applications
Source: Dis Markers. 2015 May 20;2015:973597. doi: 10.1155/2015/973597 (PMC4452852; doi:10.1155/2015/973597)
Supplement: Supplementary file 1 — Supplementary Material includes a list of primer sequences, together with gene symbols and accession numbers. This information is shown in Additional File 1. Standard curves generated for each primer are shown in Additional File 2; DNA concentrations and efficiencies are reported. All assays meet requirements of the Minimum Information for Publication of Quantitative Real-time PCR Experiments (MIQE) Guidelines; MIQE checklist is provided in Additional File 3. [file 973597.f1.pdf]

Additional File 1

Item to check	Importance	
**Experimental design**		
Definition of experimental and control groups	E	Experimental group: men with prostate cancer (PCa); control group: subjects with hystologically confirmed benign prostatic hyperplasia (BPH). The stability of candidate reference genes (2 microRNAs - miR-191, miR-25 and 1 small nucleolar RNA - SNORD48)was assessed in urine sediments after digital rectal examination (DRE).
Number within each group	E	Experimental group: 35 men with PCa; control group: 26 subjects with hystologically confirmed benign prostatic hyperplasia (BPH).
Assay carried out by the core or investigator's laboratory	D	
Acknowledgment of authors' contribution	D	
**Sample**		
Description	E	Voided urine obtained immediately after digital rectal examination (DRE) is greatly enriched in prostatic cells representing a candidate starting material for biomarker discovery in prostate cancer. Patient stratification is reported in Table 1. 18 patients presented Pathological Gleason score (GS) 6, 12 were GS 7 (3+4) and 4 were GS 7 (4+3).
Volume/mass of sample processed	D	The first 30 mL of first catch voided urine after attentive digital rectal examination were processed.
Microdissection or macrodissection	E	not applicable
Processing procedure	E	Urine samples were immediately centrifuged (2000 x g, 10 min, 4 °C). Cell pellets were washed twice with phosphate buffered saline 1x. 300 µL Lysis and Stabilization Buffer were added to the pellets.
If frozen, how and how quickly?	E	Lysates were immediately stored at -80 °C.
If fixed, with what and how quickly?	E	not applicable
Sample storage conditions and duration (especially for FFPE samples)	E	Lysates were stored at -80 °C before RNA extraction which took place no more than 2 weeks later.
**Nucleic acid extraction**		
Procedure and/or instrumentation	E	A column-based total RNA extraction Kit was used.
Name of kit and details of any modifications	E	Total RNA Extraction Kit (Norgen Biotek Corp. Ontario, Canada).
Source of additional reagents used	D	Ethanol, nuclease-free water (Sigma-Aldrich, USA)
Details of DNase or RNase treatment	E	No DNase treatment was performed

Contamination assessment (DNA or RNA)	E	
Nucleic acid quantification	E	Total RNA was quantified by Qubit RNA assay.
Instrument and method	E	Qubit 2,0 (Life technologies). For each sample, 10 µL RNA were diluted in 190 µL working solution (199 µL Reagent B + 1 µL Reagent A). After 2 minutes, stock concentrations were recorded as ng/µL (volume sample input: 10 µL).
Purity (A260/A280)	D	260/280 ratio was 1.84 [range 1.84-2.03]).
Yield	D	RNA concentration ranged from 0,5 to 14 ng/µL. No statistically significant difference in RNA yields were observed between PCa and BPH group (p > 0,05)
RNA integrity: method/instrument	E	RNA integrity was calculated with Bioanalyzer.
RIN/RQI or Ct of 3 and 5 transcripts	E	Mean RNA integrity number - RIN was 8.7 [range 8.3-9.0]
Electrophoresis traces	D	
Inhibition testing (Ct dilutions, spike, or other)	E	0,5 µL of UniSp6 spike-in control were added to the retrotranscription mix before incubation as a positive cDNA synthesis control to exclude inhibition.
**Reverse transcription**		
Complete reaction conditions	E	For each sample, RT Master Mix was prepared mixing 5X REACTION BUFFER (2 µL), ENZYME MIX (1 µL) and RNA SPIKE IN (0,5 µL). Then RNA (7,5 ng) diluted in water was added (fianl volume: 10 µL). Wells were kept 60 min at 42 °C to allow retrotranscription. Reaction was stopped raising temperature for 5 min at 95 °C.
Amount of RNA and reaction volume	E	7,5 ng of total RNA were reverse transcribed with miRCURY LNA Universal RT miR PCR, polyadenylation and cDNA synthesis kit (Exiqon). 0,5 µL of UniSp6 spike-in control were added to the retrotranscription mix (total volume 10 µL) before incubation as a positive cDNA synthesis control.
Priming oligonucleotide (if using GSP) and concentration	E	Reverse transcription reaction buffer includes universal reverse transcription primer. A polyA tail is added to the mature microRNA template. cDNA is synthesized using a PolyT primer with a 3’ degenerate anchor and a 5’ universal tag.
Reverse transcriptase and concentration	E	Enzyme Mix was shipped in 20 µL format (as part of Universal cDNA synthesis kit II Exiqon), 10 x concentrated.
Temperature and time	E	60 min at 42 °C, 5 min at 95 °C.
Manufacturer of reagents and catalogue numbers	D	miRCURY LNA Universal RT miR PCR, polyadenylation and cDNA synthesis kit (Exiqon) Cat. n. 203301
Cts with and without reverse transcription	D	
Storage conditions of cDNA	D	cDNA was stored at -20°C
**qPCR target information**		
Gene symbol	E	
Sequence accession number	E	Additional File 2
Location of amplicon	D	

Amplicon length	E	
In silico specificity screen (BLAST, and so on)	E	
Pseudogenes, retropseudogenes, or other homologs?	D	
Sequence alignment	D	
Secondary structure analysis of amplicon	D	
Location of each primer by exon or intron (if applicable)	E	
What splice variants are targeted?	E	
**qPCR oligonucleotides**		
Primer sequences	E	
RTPrimerDB identification number	D	
Probe sequences	D	
Location and identity of any modifications	E	No modifications
Manufacturer of oligonucleotides	D	LNA™ PCR Primer mix (dried down), Exiqon
Purification method	D	
**qPCR protocol**		
Complete reaction conditions	E	5 µL of SYBR Green master mix were mixed with 1 µL of primer mix and 4 µL of diluted cDNA (1:10). Thermal cycling conditions included 10 min at 95°C for enzyme activation and 45 cycles of amplification (15 sec 95°C for denaturing double stranded DNA and 1 min at 60°C for annealing/extension steps). Melting curve analysis was performed to assess amplification specificity. Each sample was run in triplicate and the results were averaged; no-template controls were included in the analysis. For each sample, LNA control primer Set were used to amplify UniSp6 spike-in positive control.
Reaction volume and amount of cDNA/DNA	E	Total reaction Volume was 10 µL, 5 µL of SYBR Green master mix were mixed with 1 µL of primer mix and 4 µL of diluted cDNA (1:10)
Primer, (probe), Mg2 , and dNTP concentrations	E	LNA™ PCR Primer mix (dried down), Exiqon. The exact composition of the Buffer is not provided by the manufacturer.
Polymerase identity and concentration	E	Hot start (included in ExiLENT SYBR® Green master mix)
Buffer/kit identity and manufacturer	E	ExiLENT SYBR® Green master mix, 20 ml (product # 203421)
Exact chemical	D	

composition of the buffer		
Additives (SYBR Green I, DMSO, and so forth)	E	SYBR Green
Manufacturer of plates/tubes and catalog number	D	Individual assays were performed on BioRad 96-Well Plates for PCR
Complete thermocycling parameters	E	Initial denaturation: 95°C for 10 minute. 45 cycles at 95°C for 15 sec, 60°C for 1 min
Reaction setup (manual/robotic)	D	Manual
Manufacturer of qPCR instrument	D	PCR reactions were performed on a Bio-Rad iCycler (BioRad, Hercules, CA).
**qPCR validation**		
Evidence of optimization (from gradients)	D	
Specificity (gel, sequence, melt, or digest)	E	Melting curves were performed at the end of each PCR run.
For SYBR Green I, Ct of the NTC	E	> 43
Calibration curves with slope and y intercept	E	
PCR efficiency calculated from slope	E	Additional File 3<br>Standard curves were generated using serial dilutions of known quantities of cDNA in triplicate.<br>Standard curves for each primer set were plotted showing Ct (y) versus log copy number of cDNA (x). The slope of the standard curve describes the efficiency of PCR, and is defined from the equation Ct=m (log Q)+c, where Ct is the threshold cycle, Q is the initial copy number and c is the intercept on the y-axis.<br>From this standard curve, information about the performance of the reaction as well as various reaction parameters (including slope, y-intercept, and correlation coefficient) can be derived.<br>The concentrations chosen for the standard curve should encompass the expected concentration range of the target in the experimental samples.
CIs for PCR efficiency or SE	D	
r2 of calibration curve	E	
Linear dynamic range	E	
Ct variation at LOD	E	
CIs throughout range	D	
Evidence for LOD	E	
If multiplex, efficiency and LOD of each assay	E	
**Data analysis**		
qPCR analysis program (source, version)	E	Bio-Rad iCycler iQTM software
Method of Ct determination	E	Baseline and threshold values were automatically determined for all reactions.
Outlier identification and disposition	E	Wells detecting RNA spike-ins (UniSp6) were compared and outlier samples (Unisp6 Ct > 30) were excluded from data analysis.
Results for NTCs	E	Not determined in the vast majority of cases, in few cases Ct > 43

Justification of number and choice of reference genes	E	This study was aimed at the selection of stable reference genes in post DRE urine sediments.
Description of normalization method	E	Ct values of miR-191, miR-25 and SNORD48 from prostate cancer (PCa) patients were normalized using BPH group as the calibrator sample. Relative expression was calculated as ΔCt (e.g.: ΔCtmiR-25 = CtmiR-25, PCa - CtmiR-25, BPH averaged).
Number and concordance of biological replicates	D	35 prostate cancer subjects were included as biological replicates.
Number and stage (reverse transcription or qPCR) of technical replicates	E	Each sample was run in triplicate and the results were averaged; no-template controls were included in the analysis.
Repeatability (intraassay variation)	E	
Reproducibility (interassay variation, CV)	D	
Power analysis	D	
Statistical methods for results significance	E	Mann-Whitney test was perfomed to verify if candidate genes were differentially expressed in the two groups.
Software (source, version)	E	GraphPad Prism 6.0 (GraphPad Software, Inc., San Diego, CA), XLSTAT (Addinsoft), BestKeeper.
Ct or raw data submission with RDML	D	

Additional File 2 - Primer details and sequence reference (available from http://www.mirbase.org and http://www.ensembl.org/).

Gene	Product name (Exiqon)	Product no.	Sequence reference	Target sequence
miR-191	hsa-miR-191-5p LNA™ PCR primer set, UniRT	204306	MIMAT0000440	CAACGGAAUCCCAAAAGCAGCUG
miR-25	hsa-miR-25-3p LNA™ PCR primer set, UniRT	204361	MIMAT0000081	CAUUGCACUUGUCUCGGUCUGA
SNORD48	miRCURY LNA™ Universal RT microRNA PCR, reference gene primer set, 200 rxns	203903	ENSG00000201823	AGUGAUGAUGACCCCAGGUAACUCUGAGUGU GUCGCUGAUGCCAUCACCGCAGCGCUCUGACC
miR-200b	hsa-miR-200b-3p LNA™ PCR	206071	MIMAT0000318	UAAUACUGCCUGGUAAUGAUGA

	primer set, UniRT			
miR-452	hsa-miR-452-5p LNA™ PCR primer set, UniRT	204301	MIMAT0001635	AACUGUUUGCAGAGGAAACUGA

Additional File 3

Standard curves were generated using serial dilutions of known quantities of cDNA in triplicate.
Standard curves for each primer set were plotted showing Ct (y) versus log copy number of cDNA (x). The slope of the standard curve describes the efficiency of PCR, and is defined from the equation Ct=m (log Q)+c, where Ct is the threshold cycle, Q is the initial copy number and c is the intercept on the y-axis.
From this standard curve, information about the performance of the reaction as well as various reaction parameters (including slope, y-intercept, and correlation coefficient) can be derived.
The concentrations chosen for the standard curve should encompass the expected concentration range of the target in the experimental samples.

dilution 1:4	1/256	1/64	1/16	1/16	1/4	1
pg DNA	30	120	480	1920	7680	30720
LOG	1,48	2,08	2,68	3,28	3,89	4,49

	miR-191			miR-25			SNORD48			miR-200b			miR-452		
1,48	33,73	34,46	33,33	33,76	35,89	34,52	34,56	33,93	35,63	34,86	35,98	35,79	32,87	34,12	34,20
2,08	31,94	27,16	31,50	33,73	32,24	33,60	36,29	34,32	32,30	32,29	35,57	31,74	33,17	33,64	31,08
2,68	30,78	33,48	30,66	31,34	32,04	30,45	31,86	32,20	31,45	29,39	30,10	29,69	31,58	31,03	33,27
3,28	27,94	28,16	27,99	31,13	30,70	29,70	28,62	27,82	30,09	27,28	27,46	27,01	28,88	29,72	29,31
3,88	24,95	25,01	25,79	24,81	26,88	25,21	26,64	27,50	27,92	27,72	27,48	27,86	27,17	27,47	29,03
4,48	25,17	22,13	23,87	24,39	26,53	24,48	27,22	25,00	25,75	23,91	24,76	22,39	24,19	25,84	25,71

Ct miR-191 slope = –3.381; E = 1.98; R2= 0,8238
Ct miR-25 slope = –3.108; E = 2.09 R2= 0,9001
Ct SNORD48 slope = –2.986; E = 2.16 R2= 0,8998
Ct miR-200b slope = –3.342; E = 1.99 R2= 0,9066
Ct miR-452 slope = –2.986; E = 2.16 R2= 0,8972

EFFICIENCY 1,98 Y = a + bx Efficiency 98%
Ct = 39,2 + (-3,38*X) 10^(-1/b)-1

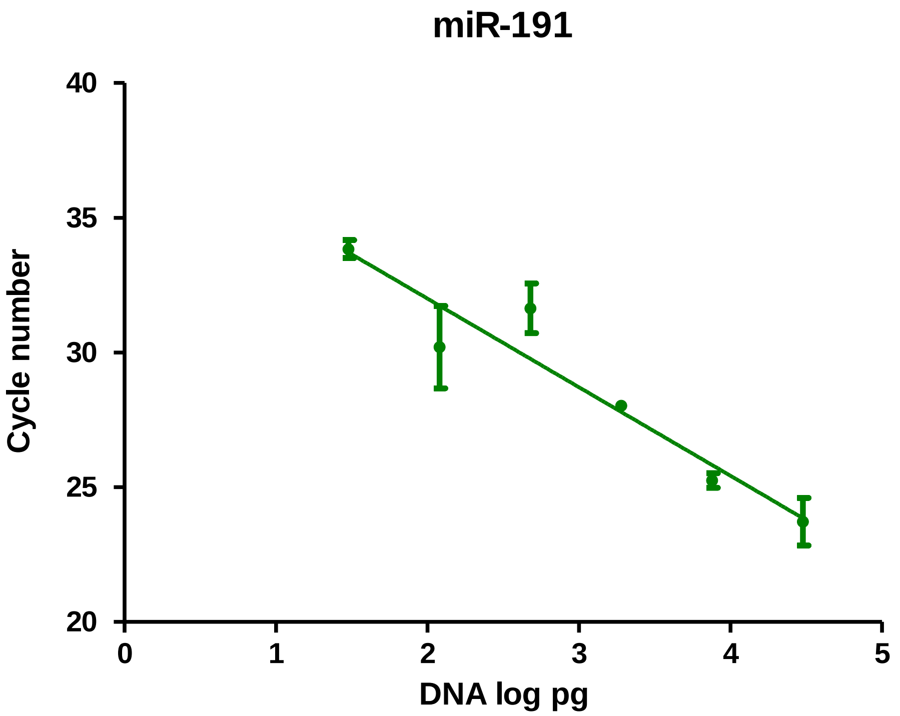

miR-191
Cycle number
40
35
30
25
20
0
1
2
3
4
5
DNA log pg

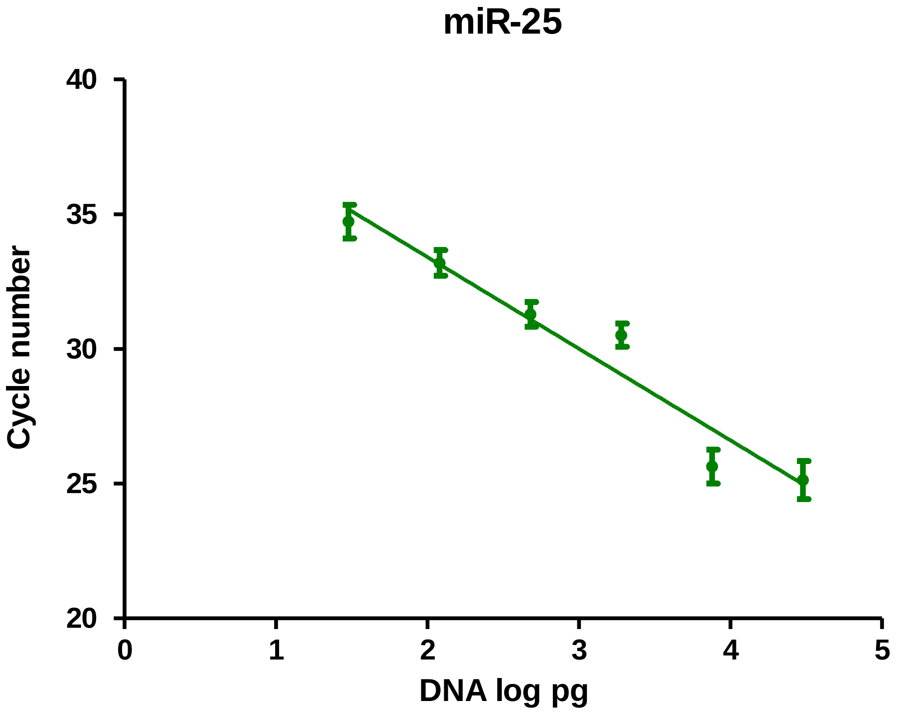

miR-25
Cycle number
40
35
30
25
20
0
1
2
3
4
5
DNA log pg

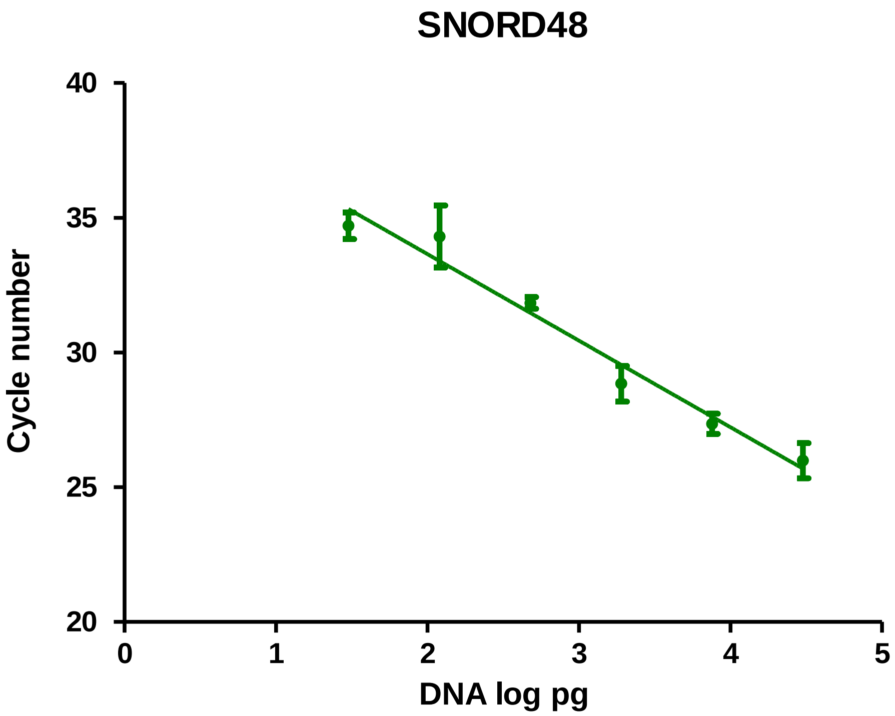
SNORD48
Cycle number
DNA log pg
40
35
30
25
20
0
1
2
3
4
5

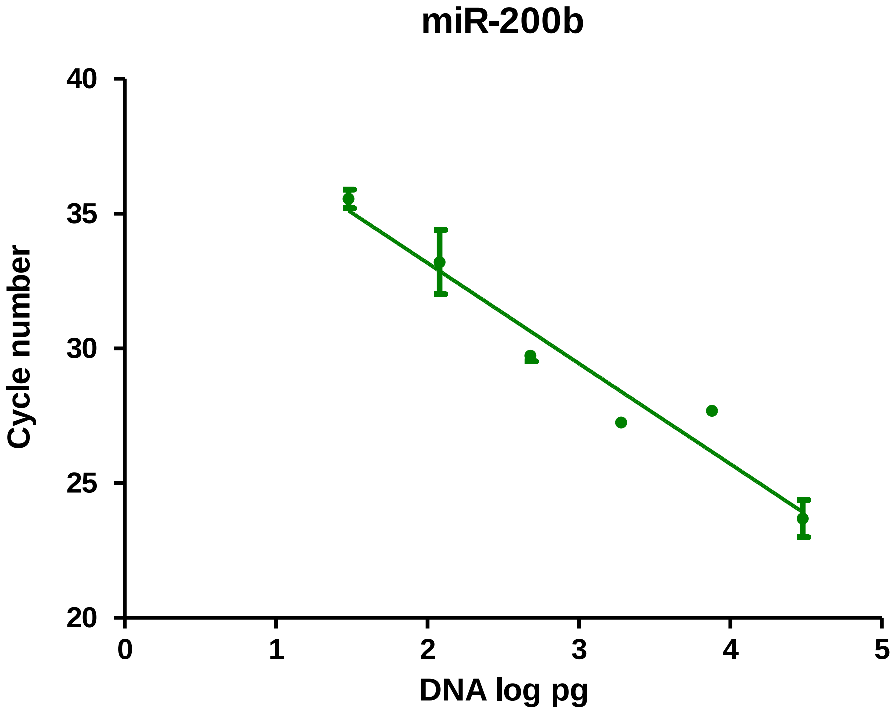
miR-200b
Cycle number
DNA log pg
40
35
30
25
20
0
1
2
3
4
5

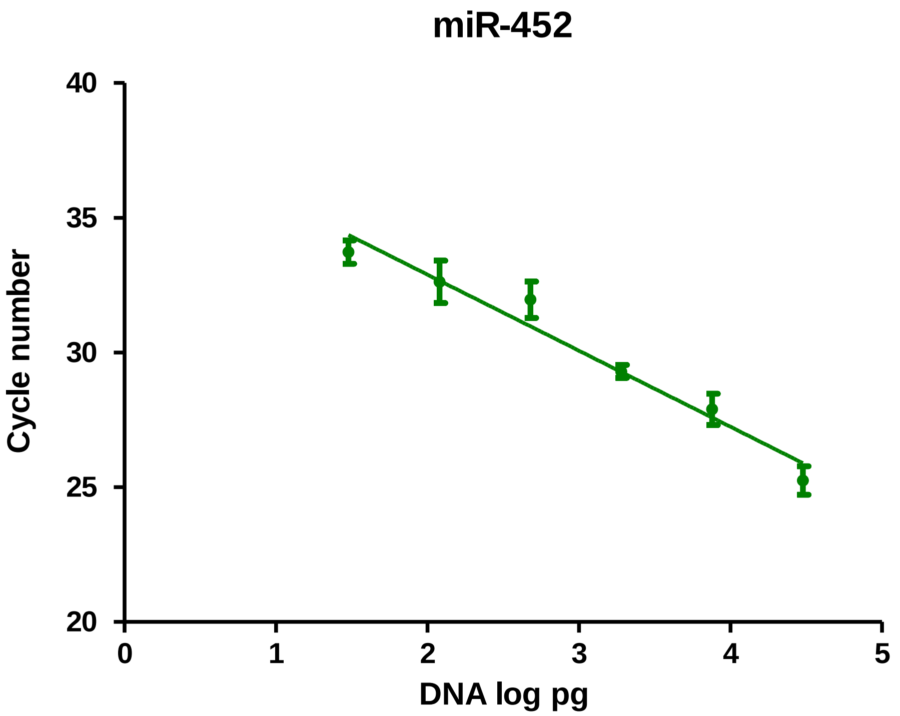
miR-452
Cycle number
40
35
30
25
20
0
1
2
3
4
5
DNA log pg
